# Chronic GPER1 Activation Protects Against Oxidative Stress-Induced Cardiomyoblast Death via Preservation of Mitochondrial Integrity and Deactivation of Mammalian Sterile-20-Like Kinase/Yes-Associated Protein Pathway

**DOI:** 10.3389/fendo.2020.579161

**Published:** 2020-10-19

**Authors:** Abdulhafiz Imam Aliagan, Ngonidzashe B. Madungwe, Nathalie Tombo, Yansheng Feng, Jean C. Bopassa

**Affiliations:** ^1^Department of Cellular and Integrative Physiology, School of Medicine, University of Texas Health Science Center at San Antonio, San Antonio, TX, United States; ^2^Department of Biomedical Engineering, University of Texas at San Antonio, San Antonio, TX, United States

**Keywords:** G protein-coupled estrogen receptor 1 (GPER1), rat cardiomyoblasts, mitochondrial function, MST1/YAP pathway, mPTP opening, mitochondrial dynamics, cell cycle

## Abstract

**Introduction:** Estrogen (17β-estradiol, E2) is well-known to induce cardioprotective effects against ischemia/reperfusion (I/R) injury. We recently reported that acute application of E2 at the onset of reperfusion *in vivo* induces cardioprotective effects against I/R injury *via* activation of its non-steroidal receptor, G protein-coupled estrogen receptor 1 (GPER1). Here, we investigated the impact and mechanism underlying chronic GPER1 activation in cultured H9c2 rat cardiomyoblasts.

**Methods:** H9c2 rat cardiomyoblasts were cultured and pretreated with the cytotoxic agent H_2_O_2_ for 24 h and incubated in the presence of vehicle (control), GPER1 agonists E2 and G1, or GPER1 agonists supplemented with G15 (GPER1 antagonist) for 48 or 96 h. After treatment, cells were collected to measure the rate of cell death and viability using flow cytometry and Calcein AM assay or MTT assay, respectively. The resistance to opening of the mitochondrial permeability transition pore (mPTP), the mitochondrial membrane potential, and ATP production was assessed using fluorescence microscopy, and the mitochondrial structural integrity was observed with electron microscopy. The levels of the phosphorylation of mammalian sterile-20-like kinase (MST1) and yes-associated protein (YAP) were assessed by Western blot analysis in whole-cell lysate, while the expression levels of mitochondrial biogenesis genes, YAP target genes, and proapoptotic genes were measured by qRT-PCR.

**Results:** We found that after H_2_O_2_ treatment, chronic E2/G1 treatment decreased cell death effect was associated with the prevention of the S phase of the cell cycle arrest compared to control. In the mitochondria, chronic E2/G1 activation treatment preserved the cristae morphology, and increased resistance to opening of mPTP, but with little change to mitochondrial fusion/fission. Additionally, chronic E2/G1 treatment predominantly reduced phosphorylation of MST1 and YAP, as well as increased MST1 and YAP protein levels. E2 treatment also upregulated the expression levels of TGF-β and PGC-1α mRNAs and downregulated PUMA and Bim mRNAs. Except for ATP production, all the E2 or G1 effects were prevented by the cotreatment with the GPER1 antagonist, G15.

**Conclusion:** Together, these results indicate that chronic GPER1 activation with its agonists E2 or G1 treatment protects H9c2 cardiomyoblasts against oxidative stress-induced cell death and increases cell viability by preserving mitochondrial structure and function as well as delaying the opening of mPTP. These chronic GPER1 effects are associated with the deactivation of the non-canonical MST1/YAP mechanism that leads to genetic upregulation of cell growth genes (CTGF, CYR61, PGC-1α, and ANKRD1), and downregulation of proapoptotic genes (PUMA and Bim).

## Introduction

Estrogen (17β-estradiol, E2) has been shown to exert protective effects against various deleterious conditions. Studies in gerbils (1), mice (2), and rats (3) revealed that female animals exhibited smaller cerebral infarct sizes than their male counterparts following carotid artery occlusion or middle cerebral artery occlusion, suggesting a neuroprotective role for estrogen. In the cardiovascular system, others and our group have provided evidence that acute pre- or post-E2 treatment can induce cardioprotective effects against ischemia/reperfusion (I/R) injury, cardiac hypertrophy, cardiac remodeling, and heart failure (4–8). Further, studies in mice revealed that E2 treatment confers renoprotective effects and ameliorates acute kidney outcomes following severe cardiac arrest (9, 10).

E2 has been shown to act via its three known estrogen receptors (ER): ERα (11), ERβ (12), or the G-protein coupled estrogen receptor 1 (GPER1) (13). Estrogen is the most abundant female sex hormone, which activates complex pathways involving genomic targets mediated through the classical receptors, ERα and ERβ (11, 12). The classical genomic pathways involve transcription of prosurvival genes facilitated by estrogen response elements (EREs) (14) and super-enhancers (15). Another genomic signaling is controlled by an indirect nuclear ER binding to DNA that is mediated by cofactors like NF-κB or AP-1 and SP-1 to exert their transcription regulation (16, 17). Classic ERs at the plasma membrane and cytosol can also mediate E2 action via non-genomic signaling, including activating kinases or binding to scaffold proteins to modulate multiple prosurvival pathways (17–19). Besides these effects mediated through its two steroidal ERs, a complementary but separate mode of rapid E2 actions have been reported that depend on agonist activation of the membrane-bound GPER1. GPER1 is highly expressed in almost all the organs, including the myocardium (13, 20), brain (21), kidney (22), and myometrium (23). Although E2's genomic mechanisms via the nuclear ERs are now well-characterized, those predominantly mediated by GPER1 activation still need to be explored. More recently, there has been a new wave of research focusing on GPER1's mechanisms of mediating estrogenic responsive effects. In fact, others and we have provided evidence that GPER1 activation with its specific agonist, G1, confers cardioprotective effects against I/R injury (20, 24, 25) via MAPK, PI-3K/Akt, and MEK/ERK/GSK3β pathways. In isolated rat hearts subjected to I/R, GPER1 activation was shown to improve functional recovery and reduce myocardial infarct size (24). Further, using isolated perfused hearts from male GPER1 knockout mice, we showed that GPER1, but not the classical ERs, plays a key role in mediating acute pre-ischemic E2-induced cardioprotection against I/R injury (4). We also showed that acute GPER1 stimulation during reperfusion elicits cardioprotective effects involving the delay of mitochondrial permeability transition pore (mPTP) opening, reduction of mitochondrial dysfunction, and mitophagy (5, 20). In intestinal crypt cells, pre-ischemic GPER1 activation has been suggested to alleviate the injury induced by I/R and improve proliferative ability of crypt stem cell by inhibiting iNOS expression (26).

In cells, an increase in the production of reactive oxygen species (ROS) is responsible for the induction of oxidative stress that is involved in the development of several diseases, including liver diseases (27, 28). Oxidative stress, such as an increase in H_2_O_2_ levels, results from an imbalance between the systemic production of ROS and a biological system's ability to clean the reactive intermediates. Disturbances in the normal redox state of cells can cause toxic effects through the production of peroxides and free radicals that damage all components of the cell, including proteins, lipids, and DNA, subsequently leading to cell apoptosis (29). The mechanism of this H_2_O_2_-induced apoptosis involves the inhibition of Bcl-2 family proteins and caspases. Pronsato's group has reported that 17β-estradiol (E2) can protect C2C12 skeletal muscle cells from H_2_O_2_-induced apoptosis by reverting PKCδ, JNK, and p66Shc activation and exerting a beneficial action over mitochondria (30, 31). Using the same model of C2C12 cell line treated with H_2_O_2_, Boland's group found that the inhibition of the antiapoptotic action of E2 was more pronounced when ER-beta was immunoneutralized or suppressed by mRNA silencing. In fact, Vasconsuelo et al. has shown that transfection of C2C12 cells with either ER-alpha siRNA or ER-beta siRNA blocked the activation of Akt by E2, and suggested differential involvement of ER isoforms depending on the step of the apoptotic/survival pathway (32). On the other hand, in cultured adipose tissue, islet, neuronal cells, and cardiomyocytes, E2 actions via GPER1 have been found to protect against H_2_O_2_-induced oxidative stress and toxicity (33). Further, E2 cytoprotection in these cells occurs independently of nuclear events or de novo protein synthesis and is mediated by rapid subcellular mechanisms, suggesting a classical and nuclear ER-independent mechanism (33). In this study, we will define the mechanism by which chronic E2-GPER1 activation induces cytoprotective effects against H_2_O_2_ deleterious effects, which are not fully understood yet.

The classical and nuclear ER, ERα, and ERβ, have been shown to localize in the mitochondria of cardiac cells (34, 35). Hence, their role in modulating mitochondrial structure and function in both normal and pathological conditions is not surprising. However, GPER1 has not been found to localize in mitochondria; nonetheless, studies have confirmed a role for GPER activation in the preservation of mitochondrial structural integrity and maintenance of function after I/R (36, 37). In fact, DNA microarray and gene set enrichment analysis (GSEA) in GPER1 KO mice revealed that GPER1's cardioprotective effects both in physiological and pathological conditions might be related to enhancements in mitochondrial function (37). Our group has also reported that acute pre- and post-ischemic GPER1 activation induces cardioprotective effects against I/R injury by protecting mitochondrial integrity and function, and reducing mitophagy (4, 5), hence, providing a premise for GPER1-induced mitochondrial protection. However, whether chronic GPER1 actions impact the mitochondria still needs to be studied. Also, acute GPER1's effects and signaling is starting to be well-documented; the mechanisms underlying chronic GPER1 actions still need further clarification. In fact, few studies have investigated the mechanisms involved in chronic GPER1 actions. Using cardiac arrest-induced global ischemia, chronic pretreatment with G1 *in vivo* has been shown to induce cardioprotective effects against I/R injury (38). Also, chronic activation of GPER1 using G1 has been shown to protect hippocampal and striatal neurons from injury following cardiac arrest and cardiopulmonary resuscitation (CA/CPR)-induced cerebral ischemia (38, 39).

Recently, accumulating literature suggests a strong cross-talk between the genomic and non-genomic GPER1's downstream pathways. GPER1 and the plasma membrane-associated estrogen receptors (mERs), mERα, and mERβ have been reported to mediate both genomic and non-genomic effects (40, 41). In breast cancer cells, GPER1 actions have been found to stimulate key regulators of the evolutionarily conserved Hippo pathway that involves the yes-associated protein 1 (YAP) and transcriptional coactivator with a PDZ-binding domain (TAZ), which are homologous transcription coactivators (40, 42). Moreover, GPER1 activation in the same cancer cell line has been shown to mediate the expression of an array of genes, including CTGF, CYR61, EDN1, and EGR1 (43–45), which are well-established YAP/TAZ target genes. This suggests that the Hippo/YAP/TAZ pathway might be a key downstream signaling pathway of GPER1 long-term actions, especially in breast cancer tumorigenesis (40). The Hippo pathway plays a critical role in cardiac development, regeneration, and disease (46, 47). Dysregulation of the Hippo pathway *in utero* can lead to various congenital cardiac abnormalities (46, 48, 49). Cardiac-specific deletion of the Hippo pathway components and overexpression of activated YAP in mouse embryos resulted in increased cardiomyocyte proliferation leading to cardiomegaly and enlarged hearts in embryos (48, 50). On the other hand, the ablation of YAP in cardiac tissue led to cardiac hypoplasia and lethality (48, 49). In fact, a new study suggests that YAP activation induces proliferation (cardiogenesis) in adult cardiomyocytes by partially reprograming them to a more fetal and proliferative state through enhancing chromatin accessibility (51). Activation of YAP, or deficiency of the Hippo pathway, has also been shown to improve cardiac tissue survival and function after myocardial infarction (46, 52, 53). However, whether GPRI1 activation induces protection against cell death via deactivation still needs further investigations.

In this study, using H9c2 rat cardiomyoblasts treated with a cytotoxic agent, H_2_O_2_, we investigated whether chronic GPER1 activation protects against H9c2 cell death by preserving mitochondrial integrity and deactivating the Hippo/YAP pathway.

## Materials and Methods

### Experimental Protocols

All protocols followed the Guide for the Care and Use of Laboratory Animals (US Department of Health, NIH) and received the UT Health Science Center at San Antonio Institutional Animal Care and Use Committee (IACUC) institutional approval.

### Animals

Adult male Sprague–Dawley rats (4–6 months old, *n* = 4) were obtained from Charles River Laboratories. The animals were housed in the animal-specific pathogen-free facility at UTHSCSA's main campus in cages with standard wood bedding and space for two rats. The animals had free access to food and drinking water *ad libitum* and a 12-h shift between light and darkness. The animals were selected randomly, and the data analysis was performed by a blinded investigator.

### Cell Culture and Treatments

H9c2 rat cardiomyoblast cell line was purchased from the American Type Culture Collection (ATCC Cat# CRL-1446, RRID: CVCL_0286). Cells were cultured in Dulbecco's modified Eagle's medium (DMEM; Invitrogen Life Technologies) supplemented with 10% fetal bovine serum (FBS; GIBCO-BRL, Grand Island, NY, USA), 100 U/mL penicillin–streptomycin and grown in an atmosphere of 5% CO_2_-95% humidified air at 37°C. The culture medium was changed every second day. Cells between passages 4 and 7 were seeded at a density of 0.5 × 10^6^ in six-well plates, or a density of 0.7 × 10^6^ in T-25 flasks and were used for experiments at 80–90% confluence (~1.5 × 10^6^ for six-well plates and ~2.8 × 10^6^ for T-25 flasks. Cells were regularly tested for mycoplasma using the Lookout Mycoplasma PCR detection kit (MP0035, Sigma). The cultured cells were exposed to H_2_O_2_ (Sigma, H1009) at a concentration of 100 μM for 24 h at 37°C. Cells were immediately washed three times with cold PBS, and the media was replaced with either serum-free DMEM plus 100 U/mL penicillin–streptomycin and 0.01% DMSO (Control, vehicle), or with 40 nM E2 (E8875, Sigma), or 1 μM G1 (Cayman, 10008933), or E2 (40 nM)/G1 (1 μM) + G15 (1 μM, Cayman, 14673), for 48 and 96 h. The cells were selected randomly, and the data analysis was performed by a blinded investigator.

### Cell Viability

Cell viability was assessed spectrofluorometrically using either a Calcein AM assay (ThermoFisher Scientific, catalog no. C3100MP) according to the manufacturer's instructions or 3-(4, 5-dimethylthiazol-2-yl)-2,5-diphenyltetrazolium bromide (MTT) assay by following standard protocols. Briefly, cells were cultured in a 96-well plate and treated with 100 μM H_2_O_2_ for 24 h, followed by the incubation in a media supplemented with the different drugs for 48 or 96 h as described above. Media containing the drug treatments were carefully aspirated, and cells were used for MTT assay or Calcein AM assay.

### Flow Cytometry Analysis

Percent live and dead cells were determined using Annexin V-PE/7-AAD Apoptosis Detection kit (BD Bioscience, BD PharMingen, catalog no. 556547) according to the manufacturer's instructions with Annexin V PE replaced with Annexin V APC. Cells treated with H_2_O_2_ were washed twice in PBS and incubated in the presence of the different drugs, as described above. After 48 h of drug treatment, cells were resuspended in 400 μL of 1x binding buffer and stained with 5 μL of APC-conjugated Annexin V (BD PharMingen, catalog no. 550475) and 5 μL 7-aminoactinomycin-D (7-AAD; BD PharMingen, catalog no. 559763) and analyzed using a BD LSR II flow cytometer (BD Biosciences, San Jose, CA, USA, UTHSCSA flow cytometry core). Cells considered viable possess intact membranes and excluded both dyes (Annexin V and 7-AAD negative); cells that are in early apoptosis are Annexin V positive and PI or 7-AAD negative, and cells that are in late apoptosis or already dead are both Annexin V and PI or 7-AAD positive.

### BrdU Labeling and Cell Cycle Analysis

For cell cycle progression analysis, cells were cultured and treated as described above. Cells were labeled with propidium iodide BrdU and analyzed by flow cytometry using the FITC BrdU Flow kit obtained from BD Biosciences following the manufacturer's instructions. Briefly, cells were pulse-labeled with 10 μM BrdU in culture medium for 30 min, trypsinized washed with PBS, fixed, and permeabilized with triton (0.25%). Incorporated BrdU in cells was exposed by DNase treatment and stained by an FITC-conjugated anti-BrdU antibody. Total DNA was stained by 7-AAD (7-amino-actinomycin D). Data were collected on a BD LSR II flow cytometer (BD Biosciences, San Jose, CA, USA, UTHSCSA flow cytometry core) and analyzed with CellQuest Pro software.

### Mitochondrial Structural Integrity

H9c2 cells pretreated with H_2_O_2_ and incubated in media containing different drugs, as described above, were processed for electron microscopy imaging to observe mitochondrial quality and morphology as described previously (54). Cells were fixed in 2.5% (wt/vol) glutaraldehyde (Fluka), at 4°C overnight. Cells were then washed with PBS and fixed in 2% (wt/vol) osmium tetroxide for 2 h at room temperature. The fixed cells were dehydrated in a graded alcohol series and embedded in Eponate 12 medium, and the blocks were cured at 60°C for 48 h. Sections (70 nm) were cut with an RMC ultramicrotome and mounted on Formvar-coated grids. The sections were double-stained with uranyl acetate and lead citrate, and finally examined and imaged with a 100CX JEOL transmission electron microscope.

### Mitochondrial Membrane Potential Measurement

MMP was assessed fluorometrically using MitoTracker Red CMXROS assay kit (ThermoFisher Scientific, catalog no. M7512) according to manufacturer's protocol. H9c2 cells were plated on coverslips for labeling and allowed to reach 70–80% confluence, after which cells were treated with or without H_2_O_2_ for 24 h and subjected to different treatments (control, G1, G1 + G15) for 48 h. Carbonyl cyanide-4-(trifluoromethoxy) phenylhydrazone (FCCP; 10 μM was used as positive control). Cells were incubated with 150 nM MitoTracker Red for 1 h at 37°C. The fluorescence intensity was measured using the ImageJ program. Data plotted were normalized on cell number.

### Detection of mPTP Opening

H9c2 cells were pretreated with H_2_O_2_ for 24 h and incubated in a media containing control [vehicle, DMSO (0.01%)], G1, or G1 + G15 for 48 h. The opening of the mPTP was assessed using a Transition Pore Assay kit (MitoProbe; Life Technologies) according to the manufacturer's instructions. Briefly, cells were incubated with 2 μM Calcein and 1 mM CoCl_2_ in Hank's Balanced Salt Solution (HBSS)/Ca^2+^ at 37°C for 15 min while protected from light. Calcein diffuses into cells passively and accumulates into the cytosol and mitochondria to liberate the highly polar fluorescent dye Calcein. CoCl_2_ can quench the cytosolic fluorescence, while mitochondrial fluorescence is maintained. Opening of mPTP instigates the release of Calcein from the mitochondria into the cytosol, which results in a reduction in fluorescence. After two washes with HBSS/Ca^2+^, the calcein fluorescence intensity of cells (~30,000 for each experiment) was detected by high-content screening at 488/530 nm using an LSR II flow cytometer (BD Biosciences).

### ATP Assay

Intracellular ATP levels in cells treated with H_2_O_2_ (24 h), followed by 48 h treatment with control (vehicle), G1, or G1 + G15 were quantified using an ATP Bioluminescence Assay kit (Roche Applied Science, catalog no. A22066) according to the manufacturer's protocol. The luminescence of the cells was measured using a plate reader. The concentration of ATP in each group was obtained using an ATP standard curve and normalized to the protein concentrations of the samples, which were determined using the BCA assay.

### Western Blot Analysis

H9c2 cells were lysed in a buffer containing (150 mM NaCl, 50 mM Tris, 5 mM EDTA, 10 mM Hepes, 0.1% octylphenyl-polyethylene glycol (IGEPAL CA-630), 0.25% sodium deoxycholate, pH 7.4, supplemented with Complete Protease Inhibitor Cocktail Tablets. Whole-cell lysates were centrifuged at 13,000 × g, for 10 min at 4°C. Equal amounts (40 μg) of proteins were loaded in each well of 4–20% Tris-glycine gels (Bio-Rad) and subjected to electrophoresis for 90 min at 125 V of constant voltage as described previously (55). Gels were transferred onto a nitrocellulose membrane by electrophoretic transfer at 90 V of constant voltage for 90 min. After transfer, the membrane was washed, blocked with 5% blocking solution and probed with the following antibodies anti-phospho-YAP (Ser127) (Cell Signaling Technology Cat# 4911, RRID:AB_2218913) 2 μg/mL, anti-YAP (Cell Signaling Technology Cat# 14074, RRID:AB_2650491) 1 μg/mL, anti-phospho-Mst1/2 (pThr183), (Sigma-Aldrich Cat# SAB4504042, RRID:AB_2665403) 2 μg/mL, anti-Mst1/2 (Cell Signaling Technology Cat# 14946, RRID:AB_2798654) 1 μg/mL, anti-Drp1 (Cell Signaling Technology Cat# 8570, RRID:AB_10950498) 1 μg/mL, anti-Mfn1 (Cell Signaling Technology Cat# 14739, RRID:AB_2744531) 1 μg/mL, anti-phospho-SAPK/JNK (Thr183/Tyr185) (Cell Signaling Technology Cat# 9251, RRID:AB_331659) 2 μg/mL, anti-SAPK/JNK (Cell Signaling Technology Cat# 9252, RRID:AB_2250373) 1 μg/mL, and anti-GAPDH (Cell Signaling Technology Cat# 2118, RRID:AB_561053) 1 μg/mL, were incubated at 4°C overnight. The immunoreactive bands were visualized using secondary Li-Cor antibodies (LI-COR Biotechnologies, Lincoln, NE): IRE 800CW goat anti-rabbit antibody (LI-COR Biosciences Cat# 926-32211, RRID:AB_621843) 0.1 μg/mL, and IRE Dye 680RD goat anti-mouse antibody, LI-COR Biosciences Cat# 926-68070, RRID:AB_10956588) 0.1 μg/mL. The band intensity was quantified using Li-Cor Odyssey® CLx Imaging System.

### Transfection

pLKO1-shYAP1 and pCMV-flag S127A YAP were gifts from Kunliang Guan (Addgene plasmid # 27368; RRID:Addgene_27368) (44), (Addgene plasmid # 27370; RRID:Addgene_27370) (56). pcDNA-Flag Yap1 was a gift from Yosef Shaul (Addgene plasmid # 18881; RRID:Addgene_18881) (57). H9c2 cells passage 4–7, at 70–80% confluence, were transfected with either pLKO1-shYAP1, or pCMV-flag-S127A YAP or pcDNA-Flag Yap1 using Lipofectamine 3000 (ThermoFisher Scientific, catalog no. L3000015) according to manufacturer's protocols.

### Isolation of Adult Cardiomyocytes

Cardiomyocytes from adult 4–6 months old (minimum 300 g) rats were isolated following the procedures described in Ref. (58). Briefly, animals were injected intraperitoneally with heparin (200 IU/kg), and 20 min later, they were anesthetized with ketamine (80 mg·kg^−1^ i.p.) and xylazine (8 mg·kg^−1^ i.p.). Hearts were then harvested and instantaneously arrested in ice-cold PBS (KCl 2 mM, KH_2_PO_4_ 1.5 mM, NaCl 138 mM, Na_2_HPO_4_ 8.1 mM) to remove excess blood. Hearts were transferred to ice-cold Tyrode's solution [NaCl 130 mM, KCl 5.4 mM, MgCl_2_ 1 mM, Na2HPO4 0.6 mM, Glucose 10 mM, Taurine 5 mM, 2,3-butanedione monoxime 10 mM, and Hepes 10 mM, pH 7.4, oxygenated with 95% (vol/vol) O2–5% (vol/vol) CO_2_], and mounted on a modified Langendorff apparatus at a constant pressure of 80 cm H_2_O. After 5 min of perfusion at 37°C with Tyrode's solution, the heart was perfused for 10 min with Tyrode's solution containing 186 U/mL Collagenase Type-2 and 0.5 U/mL Protease Type-XIV, and then washed for 5 min with a high K + buffer (KB) [KCl 25 mM, KH_2_PO_4_ 10 mM, MgSO_4_ 2 mM, glucose 20 mM, Taurine 20 mM, Creatine 5 mM, K-Glutamate 100 mM, aspartic acid 10 mM, EGTA 0.5 mM, Hepes 5 mM, and 1% (wt/vol) BSA, pH 7.2 oxygenated with 95% O_2_-5% (vol/vol) CO_2_]. After washing, the left ventricle was cut into pieces in KB solution to dissociate cells. Isolated cardiomyocytes were filtered (100-μm strainer) and centrifuged for 2 min at 1,000 × g for further use. Cardiomyocytes from each heart were divided into four groups (sham, control, G1, and G1 + G36) triplicate and cultured in 24-well plate.

### Hypoxia and Reoxygenation of Isolated Cardiomyocytes

To simulate hypoxia, substrate (glucose/serum) and oxygen deprivation, freshly isolated cardiomyocytes were resuspended in serum-free/glucose-free HEPES-buffered medium [mmol/L: NaCl 113, KCl 4.7, HEPES 10, MgSO_4_ 1.2, Taurine 30, calcium chloride (CaCl_2_) 1, pH 7.4, and 37°C]. Cardiomyocytes were incubated in a controlled hypoxic chamber, O_2_/CO_2_ incubator containing a humidified atmosphere of 1% O_2_, 5% CO_2_, and 94% N_2_ at 37°C. After hypoxia, the cells were reoxygenated in a normoxic incubator at 37°C by replacing the hypoxic media with DMEM supplemented with 10% FBS. For untreated (sham) conditions, freshly isolated cardiomyocytes were washed twice with a HEPES-buffered medium (mmol/L: NaCl 113; KCl 4.7, HEPES 10, MgSO_4_ 1.2, Taurine 30, CaCl_2_ 1, pH 7.4, and 37°C) and incubated with HEPES-buffered medium supplemented with bovine calf serum (5%) and glucose (5.5 mmol/L).

### Immunofluorescence Staining

For immunofluorescence staining, cells cultured on coverslips were pretreated with H_2_O_2_ and incubated in the different milieu as described above. Cells were fixed with 4% paraformaldehyde for 15 min and permeabilized with 0.25% Triton X-100. After blocking in 3% BSA for 30 min, slides were incubated with the first antibody diluted in 1% BSA overnight. After washing with PBS, coverslips were incubated overnight with YAP antibodies (Novus Cat# NB110-58358, RRID:AB_922796) and with the secondary antibodies Alexa Fluor 488 Goat anti-rabbit (Abcam Cat# ab150077, RRID:AB_2630356). Images were taken on a Zeiss Axiovert 200M inverted motorized fluorescence microscope (Carl Zeiss Microscope, Jena, Germany).

### RNA Extraction, cDNA Synthesis, and Real-Time PCR Amplification

Total RNA was extracted from H9c2 cells using Trizol reagent (Invitrogen) followed by DNase digestion for 10 min at room temperature with RNase-Free DNAse Set (Qiagen), and cleaned up with RNeasy Mini Kit (Qiagen). The quality of the RNA was determined by electrophoresis through agarose gels; only RNA samples with 28S:18S, rRNA ratio ≥ 2were used. Oligo-dT primer was used to target mRNAs present in the total RNA samples for conversion into cDNAs by reverse transcriptase (RT). Cleaned-up total RNA (2 μg) was reverse transcribed in a final volume of 20 μL containing 1x RT buffer, 0.5 mM dNTP Mix, 10 units of RNasin RNase inhibitor (Promega), 4 units of Omniscript RT (Qiagen), and 1 μM oligo-dT primer. Samples were incubated at 37°C for 60 min, followed by RT inactivation at 95°C for 5 min. Real-time PCR and gene-specific primers were used for quantification of TGF-β, PGC1-α, Nrf1, and YAP-responsive genes and FOXO3 responsive genes using Fast SYBR™ Green Master Mix (ThermoFisher, catalog number 4385614). The specificity of the reaction was verified by melt curve analysis. The relative quantification in gene expression was determined using the 2–ΔΔCt method (59). Using this method, we obtained the fold changes in gene expression normalized to internal control genes (β-actin, and/or GAPDH). The following primers were used for amplification

**Table:** 

**Gene**	**Forward primer (5^**′**^-3^**′**^)**	**Reverse primer(5^**′**^-3^**′**^)**
TGF-β	GACCGCAACAACGCAATCTA	AGGTGTTGAGCCCTTTCCA
PGC1-α	GATGCCAACAAGAACAAAGGT	TCTGGGGTCAGAGGAAGAGA
Nrf1	CCAAACCCACAGAGAACAGAA	TCCATGCATGAACTCCATCT
CTGF	CAAGCTGCCCGGGAAAT	CGGTCCTTGGGCTCATCA
CYR61	GTGCCGCCTGGTGAAAGAGA	GCTGCATTTCTTGCCCTTTTTTAG
ANKRD1	ATCCATGATGGTTTTTCGAGTAGAGG	GGCCTCGAGTCAGAACGTAGCTATGCGC
Bim	GCCCCTACCTCCCTACAGAC	CCTTATGGAAGCCATTGCAC
PUMA	AGTGCGCCTTCACTTTGG	CAGGAGGCTAGTGGTCAGGT
GAPDH	GCAAGTTCAATGGCACAG	CATTTGATGTTAGCGGGAT
β-actin	ATCTGGCACCACACCTTC	AGCCAGGTCCAGACGCA

### Statistical Analysis

Data presented in bar graphs are expressed as means, and error bars are the standard errors of the mean (± SEMs) for a minimum of three independent trials (*n* ≥ 3). Comparisons were conducted using the one-way ANOVA with Tukey's corrections for multiple comparisons, where appropriate, using Prism 8 (Graphpad Software). A difference of *P* < 0.05 was considered to be statistically significant.

## Results

### Chronic GPER1 Activation Induces Cytoprotective Effects Against H_2_O_2_-Induced H9c2 Cell Death

**Figure 1:**
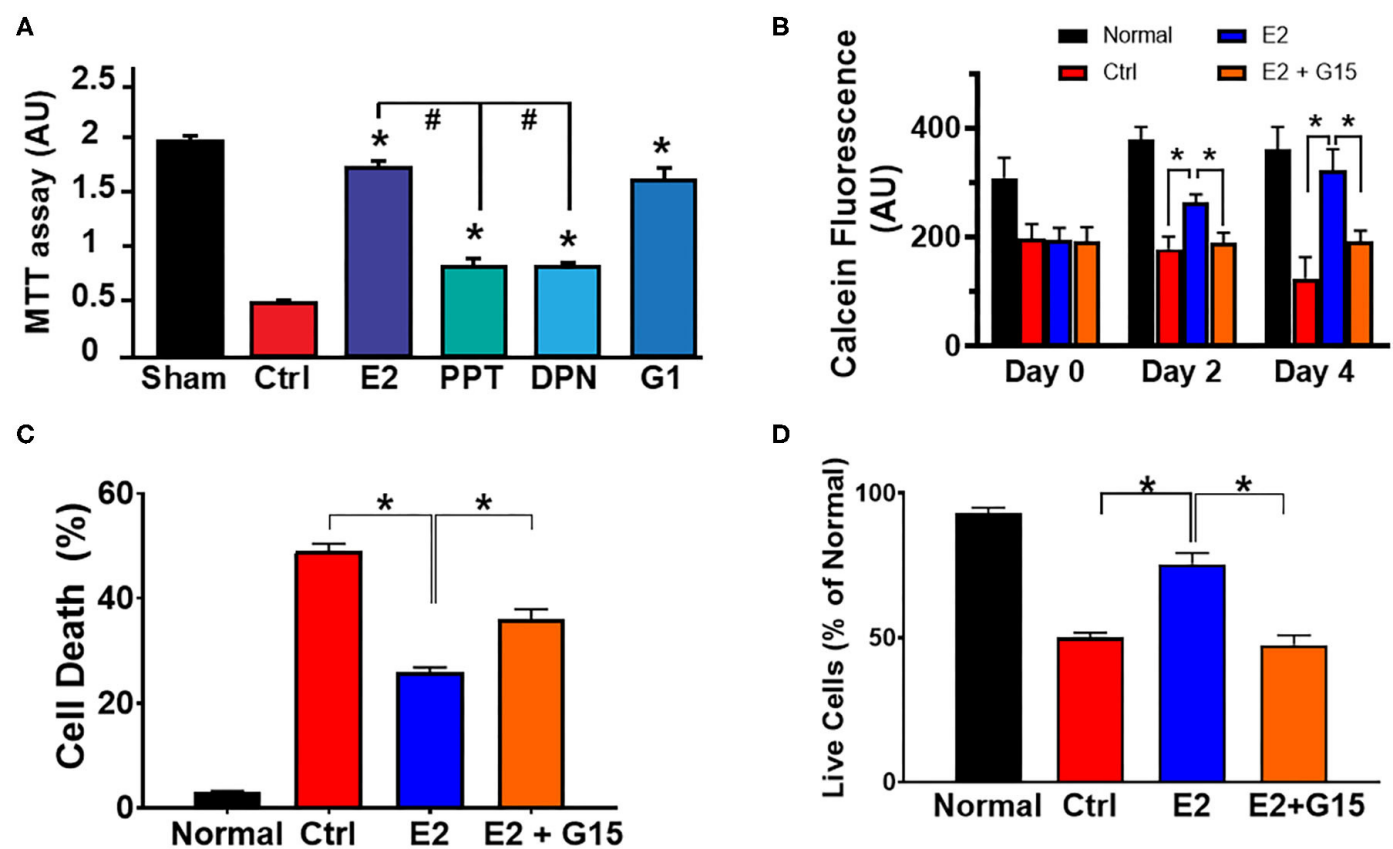
Chronic G protein-coupled estrogen receptor 1 (GPER1) activation induces cytoprotective effects in H9c2 cells. **(A)** Bar graph showing the cell viability of H9c2 cardiomyoblasts after H_2_O_2_ insult, and treatment with estrogen (17β-estradiol; E2) and specific agonists for estrogen receptor (ER)α (PPT), ERβ (DPN), and GPER1 (G1). Note that G1 cytoprotective effect is similar to E2. **(B)** Bar graph showing the Calcein AM viability assay on normal H9c2 cells at 24 h post H_2_O_2_ treatment (day 0) and after chronic treatment with either control media (Ctrl; red bars), E2 (40 nM, blue bars), or E2 (40 nM) + GPER1 antagonist (G15) (1 μM) (E2 + G15; orange bars) for 48 h (day 2) or 96 h (day 4). Values are expressed as means ± standard error of the mean (SEM) of five independent experiments *P < 0.001, *n* = 5/group, nine wells for each. Note that E2 treatment for 48 or 96 h increases in the fluorescence intensity compared to the control and E2 + G15 indicating an increase in cell viability of cells treated chronically with E2. **(C)** Bar graph showing a reduction in cell death by apoptosis (Annexin V+/PI+) in E2-treated cells compared to control (Ctrl, vehicle) and E2 + G15-treated cells. Values are expressed as means ± SEM *P < 0.05. **(D)** Bar graph showing an increase in cell viability [3-(4, 5-dimethylthiazol-2-yl)-2,5-diphenyltetrazolium bromide (MTT) assay] in the chronic E2 group compared to the control and E2 + G15. Values are expressed as means ± SEM of five independent experiments *P < 0.05, *n* = 5/group, nine wells for each. * Compared to control, # compared to E2-treated group.

E2 has been shown to act via its three known estrogen receptors (ERs): ERα, ERβ, or GPER1. We determined the influence of each ER against H_2_O_2_-induced cytotoxic effects using cultured H9c2 cardiomyoblasts. We found that E2 as well as all the ER-specific agonists, PPT (for ERα), DPN (for ERβ), and G1 (for GPER1) increased the level of live cells compared to control (Figure 1A). However, the level of live cells in the E2-treated group was significantly higher than that of PPT and DPN groups, but E2 effects were similar to G1, suggesting that the E2-induced increase in cell survival might be mainly mediated through GPER1. We, therefore, evaluated the cytoprotective effects of GPER1 activation in chronic E2 treatment against the harmful effects of oxidative stress on H9c2 cardiomyoblasts. To this end, H_2_O_2_ pretreated cells were incubated in the presence of vehicle (control), E2, or E2 + G15 (G15 is a GPER1 selective antagonist) after 48 and 96 h. We found that chronic E2 treatment increased cell viability measured by Calcein AM assay compared to control (Figure 1B). Since these chronic effects of E2 were similar after 48 and 96 h, we chose a 48-h time point for further experiments. We also found that E2 treatment reduced cell death induced by H_2_O_2_ treatment compared to control. In fact, the rate of cell death that was 49 ± 2% in the control group (vehicle) was decreased to 25 ± 1.5% in E2-treated group (Figure 1C). Similar results were obtained with cell viability (Figure 1D) in which the level of live cell was increased in the E2-treated group (75 ± 4%) vs. control (50 ± 2%). However, all these E2 protective effects were antagonized by cotreatment with G15. In fact, when E2 was cotreated with G15 (E2 + G15 group), the levels of cell death were increased to 36 ± 2%, similar to the control group (49 ± 2%), and the cell viability was decreased from the E2 group (75 ± 4%) compared to the E2 + G15 group (47.5 ± 3%).

Together, these results suggest that chronic E2 treatment induces cytoprotective effects against H_2_O_2_-induced H9c2 cell death through GPER1 activation.

### Chronic GPER1 Activation Protects Against H_2_O_2_-Induced Mitochondrial Dysfunction in H9c2 Cells

**Figure 2:**
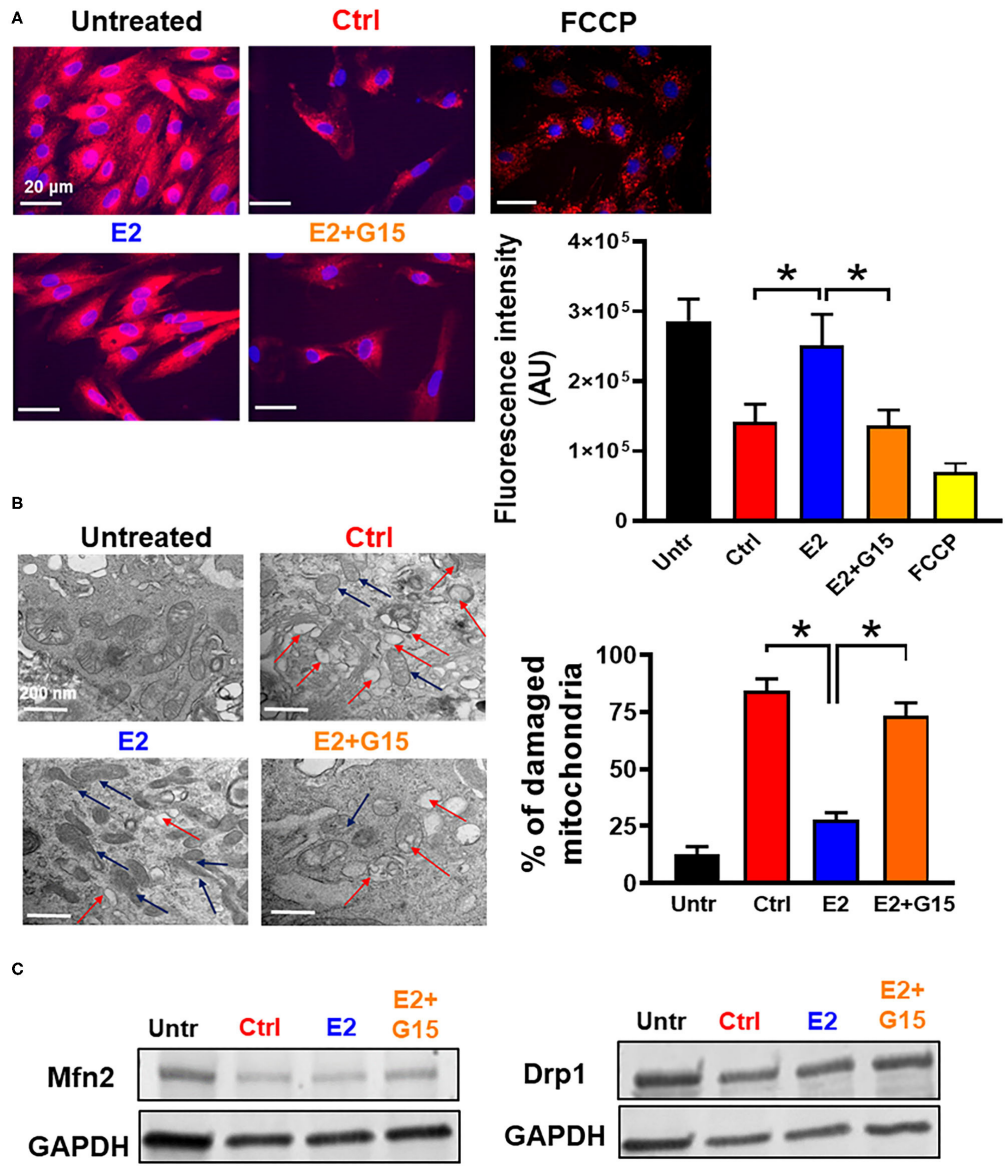
Chronic GPER1 activation preserves mitochondrial structure and function. **(A)** *Left*: Representative fluorescent microscope images used to measure mitochondrial membrane potential using the MitoTracker Red dye in H9c2 cells treated with H_2_O_2_ followed by incubation in control (Ctrl), E2 (blue), and E2 + G15 (orange), *n* = 5/group, nine wells for each. Red color denotes MitoTracker Red staining. FCCP (yellow) was used as positive control. *Right*: The relative fluorescence intensities of MitoTracker Red quantified on a per-cell basis in H9c2 cells treated as described above. Values are expressed as means ± SEM of five independent experiments *P < 0.001. **(B)** *Left*: Microscopy images of H9c2 cells showing damaged mitochondria cristae in cells treated with Ctrl, and E2 + G15 compared to E2-treated cell mitochondria. *Right*: Bar graph showing percentage of damaged mitochondria in each group. Fragmented or disrupted cristae with empty spaces (in the matrix) were considered damaged mitochondria, while mitochondria with dense continuous cristae were considered as good or undamaged. A minimum of 100 mitochondria were counted in each group. Values are expressed as means ± SEM of five independent experiments *P < 0.05, *n* = 5/group **(C)** Representative immunoblots showing no change in the levels of mitochondrial fission protein (Drp1) and mitochondrial fusion protein (Mfn2) in all the three groups Control (vehicle), E2, and E2 + G15.

The cytotoxic agent H_2_O_2_ treatment has been shown to induce mitochondrial depolarization, increase mitochondrial calcium overload, alter ATP synthase activity, and affect mitochondrial dynamics (60, 61). Therefore, we tested whether chronic GPER1 actions can restore mitochondrial function after H_2_O_2_ insult. To this end, we measured MMP and unveiled the mitochondrial structural integrity in H9c2 cells pretreated with H_2_O_2_ followed by treatment with vehicle (control), E2, or E2 + G15. We found that chronic E2 treatment prevented H_2_O_2_-induced dissipation of MMP. In fact, the control mitochondria displayed a reduction in fluorescence intensity of MitoTracker Red compared to H_2_O_2_-untreated cells (141,173 ± 26,198 vs. 286,448 ± 31,520). However, chronic E2-treated mitochondria exhibited higher fluorescence intensity, almost similar to the H_2_O_2_-untreated cell mitochondria (250,912 ± 45,145) (Figure 2A). This E2-induced higher fluorescence intensity was prevented by cotreatment with G15. These results suggest that chronic E2 treatment preservation of the MMP is mediated through GPER1 activation.

We also defined the effect of chronic GPER1 activation on the mitochondrial structure of H9c2 cells treated with H_2_O_2_. Observation of electron microscopy images of treated cells in each group showed that after H_2_O_2_, 84 ± 5% of the mitochondria from control-treated cells were damaged and characterized with smaller, ruptured, and fragmented cristae morphology compared to H_2_O_2_-untreated in which mitochondria cristae were mostly normal and not disrupted (Figure 2B). However, after H_2_O_2_ treatment, cells incubated in the presence of E2 had only displayed 28 ± 3% damaged mitochondria. In comparison, cell cotreatment with E2 + G15 exhibited 73 ± 6% damaged mitochondria. This data suggest that selective inhibition of GPER1 abridges the preservation mitochondrial integrity and function effects induced by E2 treatment.

We finally determined whether chronic activation of GPER1 preservation of mitochondria integrity and function was related to increased fission and fusion. We, therefore, measured the levels of fission proteins, dynamin-related protein 1 (Drp1), and Mitofusin 2 (Mfn2), which regulates fusion. We observed no changes in both Drp1 and Mfn2 protein levels (Figure 2C) in the E2-treated group compared to the control (vehicle), suggesting that chronic E2 preservation of mitochondrial structure and function is not via increased mitochondrial dynamics.

Together, these results indicate that chronic E2 treatment preserves against H_2_O_2_ insult increase in mitochondrial cristae damage, which presumably is responsible for the increase in mitochondrial membrane potential via GPER1 activation.

### Chronic GPER1 Activation Inhibits mPTP Opening After H_2_O_2_ Insult

**Figure 3:**
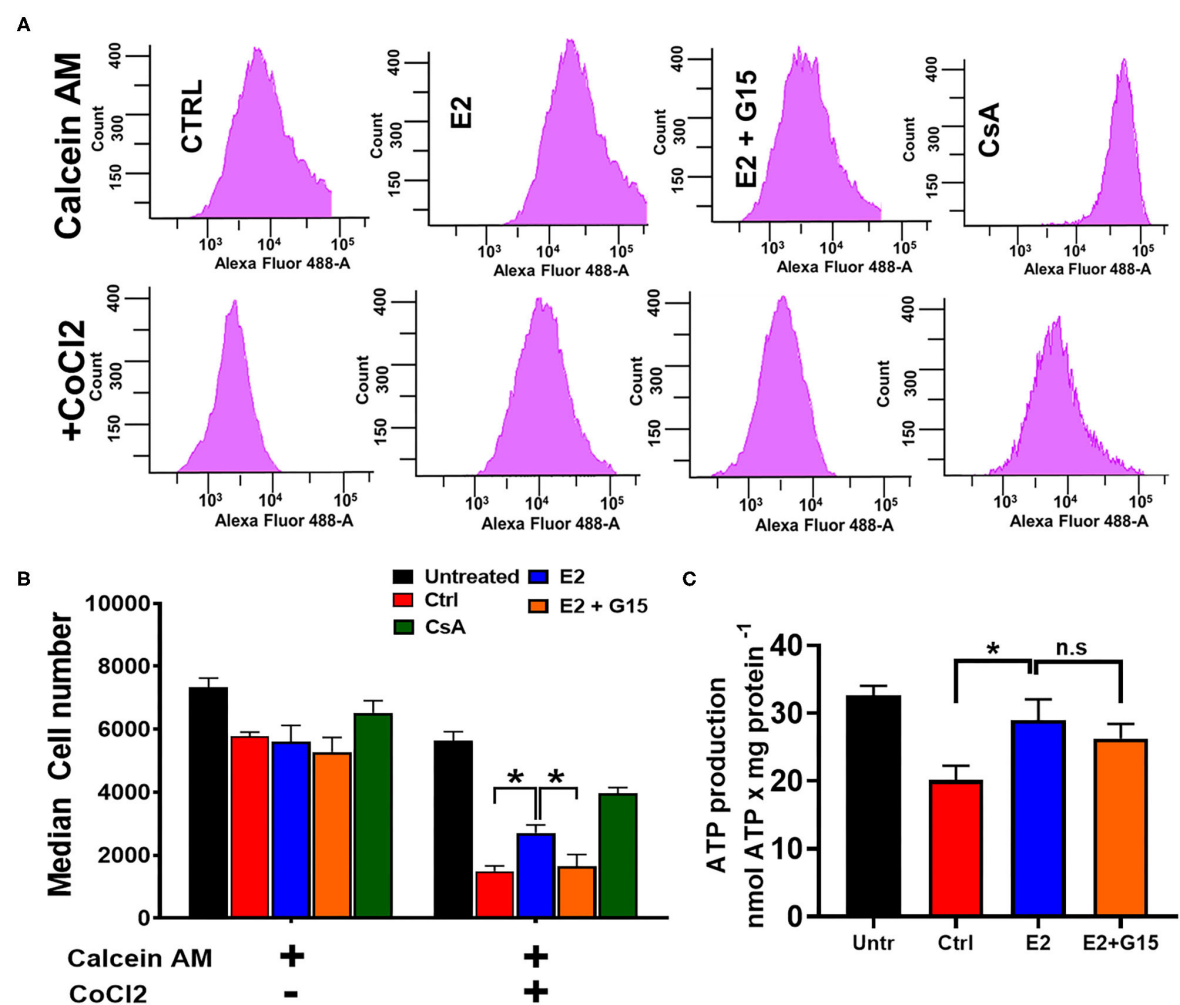
Chronic GPER1 protects against H_2_O_2_-induced mitochondrial permeability transition pore (mPTP) opening. **(A)** Representative flow cytometry histograms of calcein fluorescence in the absence of CoCl_2_ (top) and in the presence of CoCl_2_. **(B)** Graph showing calcein fluorescence intensity in H9c2 cells treated with calcein/cobalt indicating the degree of mPTP activation. The graph shows no difference in fluorescence in all groups when treated with calcein only. However, the calcein fluorescence intensity is decreased in cells treated with control (red bars) and E2 + G15 (orange bars) compared to E2 group (blue bars) after calcein + CoCl_2_ treatment. Cyclosporine A (1 μM) (green bars) was used as a positive control. Values are expressed as means ± SEM *P < 0.05, *n* = 6/group. **(C)** Bar graph showing an increase in luminescence (ATP production) in cells treated with E2 and E2 + G15 compared with the control. Note that there is no difference in ATP production in the E2 and E2 + G15 groups, suggesting a mechanism independent of GPER1. Values are expressed as means ± SEM of five independent experiments *P < 0.05, *n* = 5/group.

We further assessed the impact of the regulation of the mitochondrial permeability transition pore (mPTP) opening in the mechanism of chronic GPER1 activation. The mPTP opening is a well-known effector that mediates cell death by apoptosis and necrosis (62, 63). H9c2 cells were pretreated with H_2_O_2_ and incubated in the presence of control (vehicle), E2, E2 + G15, or Cyclosporine A (CsA). The mPTP opening was measured using the Calcein-CoCl_2_ assay. To confirm the specificity of this assay to the mPTP opening, we measured the calcein fluorescence intensity (CFI) in H_2_O_2_-treated H9c2 cells in the presence of CsA, a known inhibitor of the mPTP opening. We found that CsA treatment dramatically increased the CFI when compared to control. We observed that the CFI was drastically reduced in the vehicle-treated cells (Control) compared to H_2_O_2_-untreated cells, which indicate that mPTP opening was more active after H_2_O_2_ treatment vs. the untreated (Figures 3B,C). However, the CFI was much higher in chronic E2-treated cells compared to the control (Figures 3B,C). In addition, cotreatment with E2 + G15 displayed a much-reduced CFI compared to E2-treated cells and similar to the control group. These results indicate that chronic E2 treatment induces inhibition of mPTP opening via activation of GPER1.

In yeast, H_2_O_2_ treatment resulted in a decrease in ATP production in response to oxidative stress (64). We, therefore, sought to determine whether chronic E2 treatment following H_2_O_2_ insult in H9c2 cells enhances mitochondrial ATP production. We observed that after H_2_O_2_ treatment, control (vehicle)-treated cells decreased the levels of ATP production when compared with H_2_O_2_-untreated cells (32.6 ± 1.43 nmol/mg^−1^ in untreated compared to 20.102 ± 2.135 nmol/mg^−1^ in the Control group) (Figure 3C). Consistently, after H_2_O_2_ treatment, chronic E2 treatment increased the levels of ATP production compared to the control (28.986 ± 3.06 nmol/mg^−1^ vs. 20.102 ± 2.135 nmol/mg^−1^). However, cotreatment with E2 + G15 did not significantly change the levels of ATP production (27.14 ± 2.168 nmol/mg^−1^) vs. the E2-treated group (Figure 3C) suggesting that E2 induces preservation of the mitochondrial structure via GPER1 activation, and this effect is not associated with the increase in the rate of ATP production indicating improvement of mitochondrial function.

Together, these results indicate that chronic GPER1 activation-induced preservation of the mitochondrial integrity is associated with the inhibition of mPTP opening.

### Chronic GPER1 Activation After H_2_O_2_ Treatment Does Not Induce Mitochondrial Biogenesis

**Figure 4:**
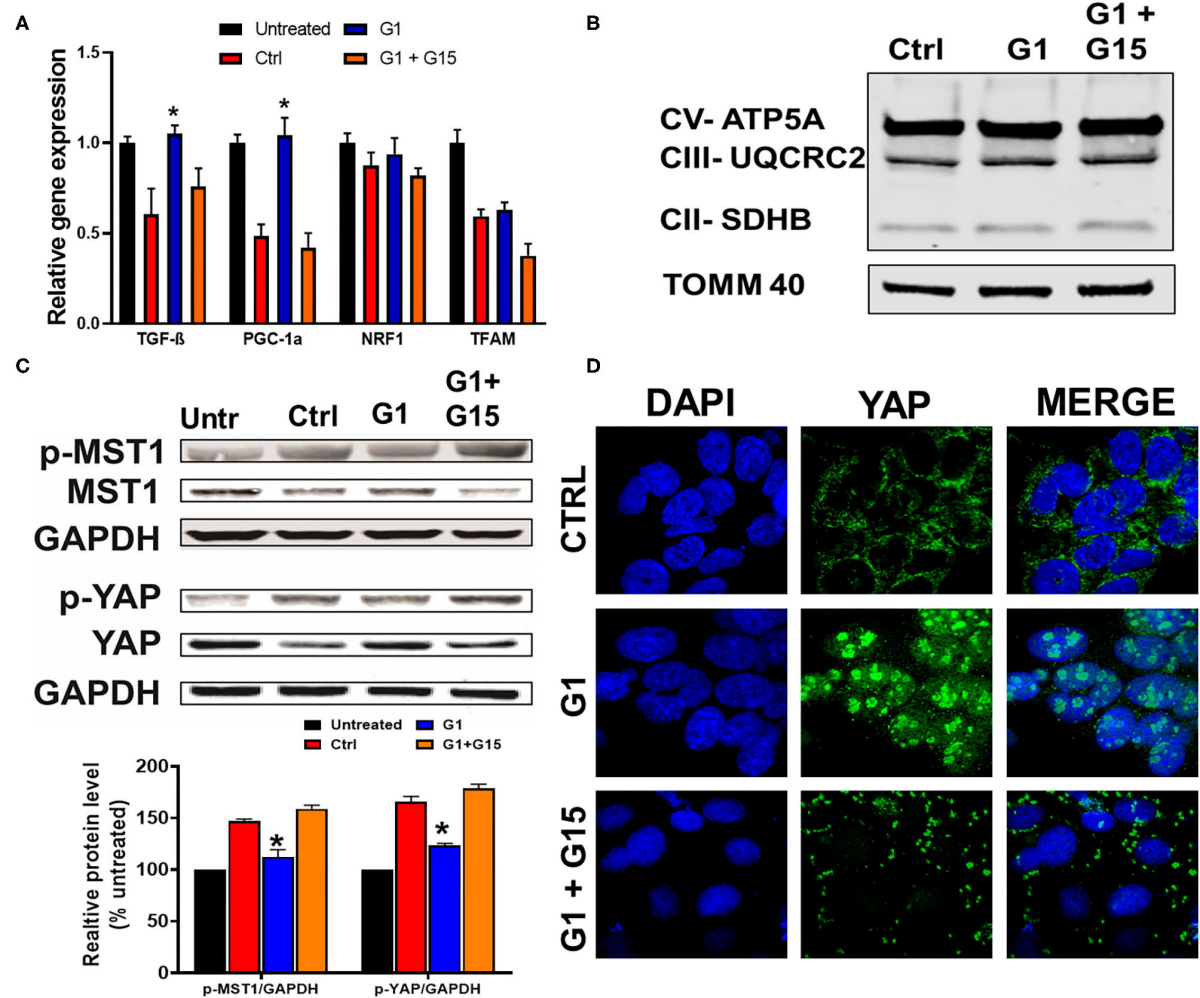
Chronic GPER1 does not impact mitobiogenesis, but deactivates the Hippo/YAP pathway. **(A)** qRT-PCR analysis of mitochondrial biogenesis genes (PGC1-α and TGF-β) in H9c2 cells after chronic treatment with control (Ctrl), G1, and G1 + G15. Error bars indicate SEM. *P < 0.05, one-way ANOVA with Tukey posttest, *n* = 6 in each group. β-Actin was used as an internal control. Note an increase in both PGC1-α and TGF-β mRNA expression levels, and no change in mitochondrial transcription factor A (TFAM) and nuclear respiratory factor 1 (Nrf1) expression levels in the G1-treated group compared to the control and G1 + G15. **(B)** Immunoblots of mitochondrial fractions showing no changes in the protein levels involved in the mitochondrial oxidative phosphorylation after G1 treatment compared to the control and G1 + G15. **(C)** *Top*: Immunoblots showing a reduction in phosphorylated mammalian sterile-20-like kinase (p-MST1) and phosphorylated yes-associated protein (p-YAP) levels in the G1-treated cells compared to the Ctrl and G1 + G15 cells. *Bottom*: Bar graph (normalized to untreated) showing a reduction in p-MST1 and p-YAP in the G1-treated cells. Values are expressed as means ± SEM of five independent experiments *P < 0.05, *n* = 5/group. **(D)** Confocal microscopy images of H9c2 cells treated with control (Ctrl), G1, or G1 + G15, for 48 h and labeled with DAPI (blue) and anti-YAP (green) as well as the overlay of both signals. Note the increase in YAP–DAPI colocalization in G1-treated cells compared to Ctrl and G1 + G15.

Since we established that most of the chronic cytoprotective effects of E2 were mediated by GPER1 activation, we, therefore, proceeded to use the selective GPER1 agonist, G1, for our other experiments. We found that chronic GPER1 activation reduces mitochondrial dysfunction caused by H_2_O_2_ treatment. We determined whether chronic GPER1 activation protects the mitochondrial structural integrity by favoring mitochondrial biogenesis. To this end, pretreated cells with H_2_O_2_ were incubated in a no-serum media in the presence of vehicle (control), G1, or G1 + G15. We measured the mRNA levels of peroxisome-proliferator-activated receptor γ coactivator-1α (PGC-1α), which is a master regulator of mitochondrial biogenesis (65) and TGF-β1 that are negatively regulated by PGC-1α (66), as well as nuclear respiratory factor 1 (Nrf1), which is known to activate mitochondrial transcription factor A (TFAM) (67). We observed that the mRNA levels of PGC-1α and TGF-β1 were increased in G1-treated cells compared to the control, and cotreatment of G1 with G15 prevented these G1 effects (Figure 4A). However, we did not observe any differences in the mRNA levels of Nrf1 or TFAM in all the groups (Figure 4A). We further measured the levels of OXPHOS proteins (CII-SDHB, CIII-UQCR2, and CV-ATP5A), known as transcriptional targets of PGC-1α. Consistently, we did not observe any differences in the levels of OXPHOS proteins among the three different treatment groups (Figure 4B). However, this surprising result was not in conformity with the increase in the TGF-β1 mRNA levels observed in the G1-treated group compared to the control, and the G1 + G15 groups (Figure 4A). We, therefore, conclude that chronic GPER1 activation following H_2_O_2_ insult does not impact the mitochondrial biogenesis process.

### Chronic GPER1 Activation Negatively Regulates the Hippo/YAP Pathway

**Figure 5:**
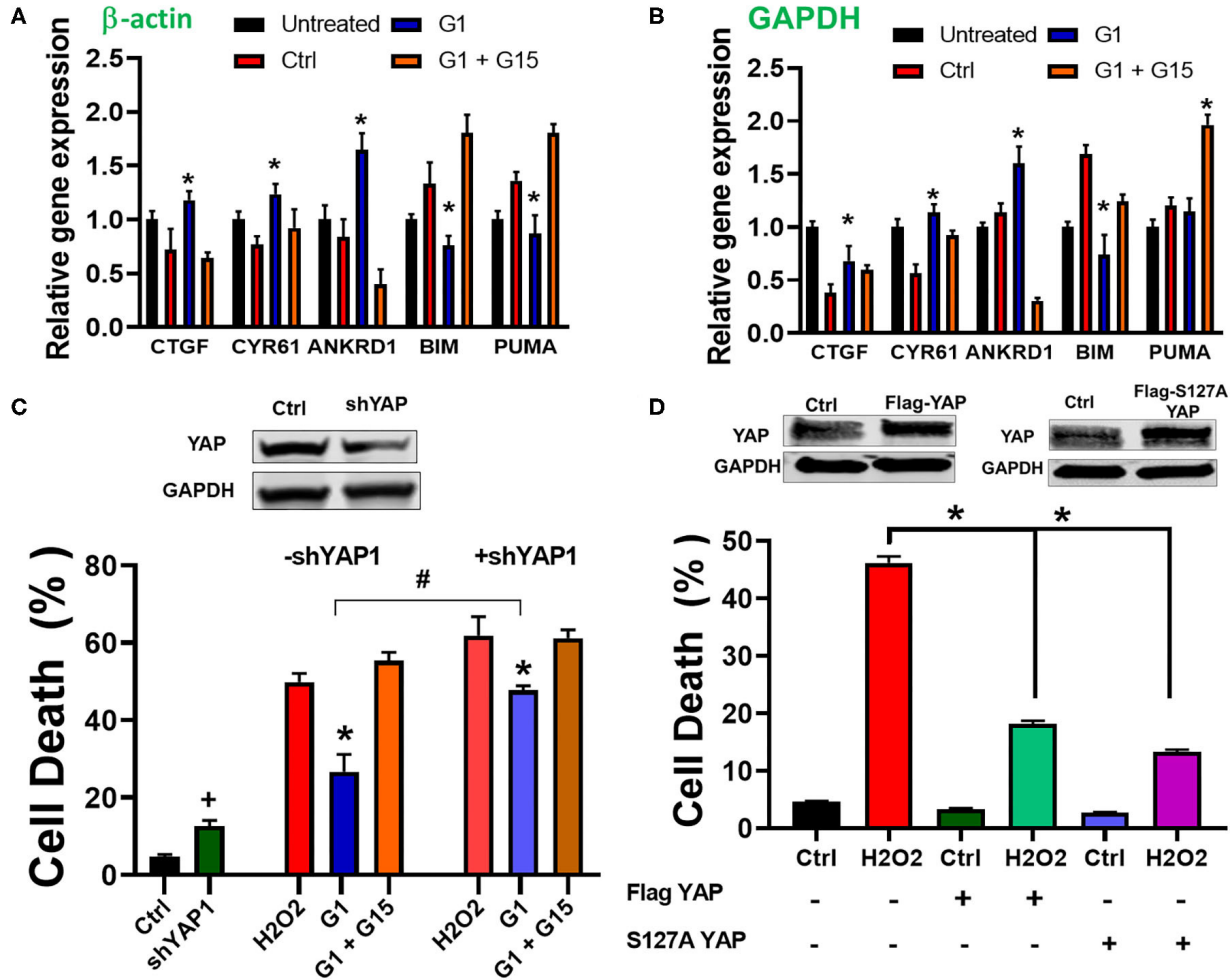
Chronic GPER1 activation increases expression of YAP target genes. **(A)** qRT-PCR analysis of YAP-responsive genes in H_2_O_2_-treated H9c2 cells after incubation with control (Ctrl, vehicle), G1, or G1 + G15 using β-actin as an internal control. **(B)** Using GAPDH as an internal control. Note that G1 treatment increases the expression levels of CYR61, CTGF, and ANKRD1 mRNA, but reduces the expression of FOXO3-responsive genes (Bim and PUMA). *N* = 6/group. Error bars indicate SEM. *P < 0.05, one-way ANOVA with Tukey post-test. **(C)** Top: immunoblot showing a reduction in YAP protein levels following knockdown by shRNA. Bottom: Bar graph showing an increase in cell death by apoptosis (Annexin V+/PI+) in YAP knockdown cells and after H_2_O_2_ treatment, followed by the incubation in the three different conditions. Note that the rate of cell death in YAP knockdown + E2 treatment was increased compared to E2 only. Values are expressed as means ± SEM *P < 0.001, *n* = 6/group, ^**#**^*P* < 0.001, *n* = 6/group vs. E2, and ^**+**^*P* < 0.05 vs. control plasmid *n* = 6/group. **(D)** Top: Immunoblots showing an increase in YAP protein levels following overexpression of Flag-YAP or S127A Flag-YAP. *Bottom*: Bar graph showing a decrease in cell death by apoptosis (Annex in V +/PI +) in YAP-overexpressed group compared to control. Note that after H_2_O_2_ treatment, the rate of cell death is reduced in YAP-overexpressing cells compared to PCMV6 plasmid (control). Values are expressed as means ± SEM *P < 0.001 *n* = 6/group.

The Hippo pathway can be regulated by hormonal signals that act through G-protein-coupled receptors (68), e.g., estrogen through GPER1 (40). Therefore, we tested whether the increase in the cells' survival induced by chronic treatment with the GPER1 agonists is mediated through the Hippo/YAP pathway. To this end, we compared the levels of phosphorylated MST1 (p-MST1) and YAP (p-YAP) in the control, G1, or G1 + G15 cells pretreated with an H_2_O_2_ agent. Western blot analysis revealed that the levels of p-MST1 and p-YAP were reduced in the G1-treated group compared to the control, and this G1 effect was abolished by the addition of G15 (Figure 4C). We also conversely found that the protein levels of both MST1 and YAP were increased in G1-treated cells compared to the control. Here, also the G1 effect was prevented by G15. Phosphorylation of YAP results in YAP−14–3–3 binding and cytoplasmic retention (69). In the contrary, non-phosphorylated YAP translocates to the nucleus, where it binds to transcriptional enhanced associate domain (TEAD) protein family to stimulate expression of cell growth and survival genes (44). We, therefore, determined whether G1 treatment increases YAP nuclear translocation. Using the immunocytochemistry approach, we observed that YAP expression was dispersed in the cytoplasm of control-treated cells (Figure 4D), while upon G1 treatment, YAP signal increasingly colocalized with DAPI (a dye that stains nucleic acids). Cotreatment of G1 with G15 prevented the nuclear translocation of YAP and enhanced its cytoplasmic retention (Figure 4D). Further, we measured the mRNA levels of YAP-responsive genes (CTGF, CYR61, and ANKRD1), and we found that chronic G1 treatment increased the transcription of CTGF, CYR61, and ANKRD1 (Figures 5A,B) using β-actin (Figure 5A) or GAPDH (Figure 5B) as an internal control. These results indicate that chronic GPER1 activation activates YAP-mediated transcription of prosurvival genes. Since MST1 has been shown to trigger FOXO3 nuclear translocation leading to apoptosis (70), we measured the mRNA levels of PUMA and Bim, which are FOXO3-responsive genes after chronic G1 treatment. Our results show that both PUMA and Bim mRNAs were significantly increased in the control group of cells pretreated with H_2_O_2_ and that chronic G1 treatment decreased the levels of these two proapoptotic genes (Figures 5A,B).

To confirm the involvement of YAP in the mechanism of chronic GPER1 action, we determined whether knockdown of YAP in cells affects the G1-induced cytoprotective effects. To this end, H9c2 cells were transfected with a plasmid containing shRNA against YAP1 (pLKO1-shYAP1). The knockdown efficiency was determined by Western blot analysis (Figure 5C). We observed that knockdown of YAP increased cell death in H9c2 cells (4.7 ± 0.53% in control plasmid vs. 12.63 ± 1.46% in pLKO1-shYAP1 transfected cells) (Figure 5C). However, the level of cell death in control plasmid treated cells was increased compared to YAP knockdown cells after treatment with control (vehicle) (48.83 ± 2.26% vs. 61.94 ± 4.82%), and in G1 + G15 (52.36 ± 3.2% vs. 61.23 ± 2.15%), in control-treated vs. pLKO1-shYAP1 treated, respectively. We also found that the rate of cell death in G1-treated cells was increased in YAP knocked down cells compared to the control (26.7 ± 4.43% vs. 47.67 ± 3.82%, respectively). This result suggests that the deletion of YAP increases cell's susceptibility to H_2_O_2_-induced cell death. We also determined whether the overexpression of YAP alone or the S127A mutant YAP, which constitutively remains in the nucleus and is transcriptionally more active (71), protects H9c2 cells from H_2_O_2_-induced cell death. We found that cells overexpressing either YAP or S127A YAP were resistant to H_2_O_2_-induced cell death (Figure 5C). In fact, while cells transfected with control (PCMV6) plasmid and treated with H_2_O_2_ resulted in 48.4 ± 3.3% cell death, those overexpressing YAP or S127A YAP resulted only in 17.2 ± 2.5% and 13.4 ± 1.6% cell death, respectively (Figure 5D). This result indicates that the regulation of YAP influences H9c2 cell death induced by H_2_O_2_ insult.

Together, these results indicate that chronic GPER1 actions are mediated by regulation of the downregulation of the Hippo/YAP pathway that includes the cytosolic accumulation of YAP and its translocation in the nucleus where it promotes the upregulation of prosurvival genes and the downregulation of proapoptotic genes.

### Chronic GPER1 Activation Reduces MST1–JNK Signaling

**Figure 6:**
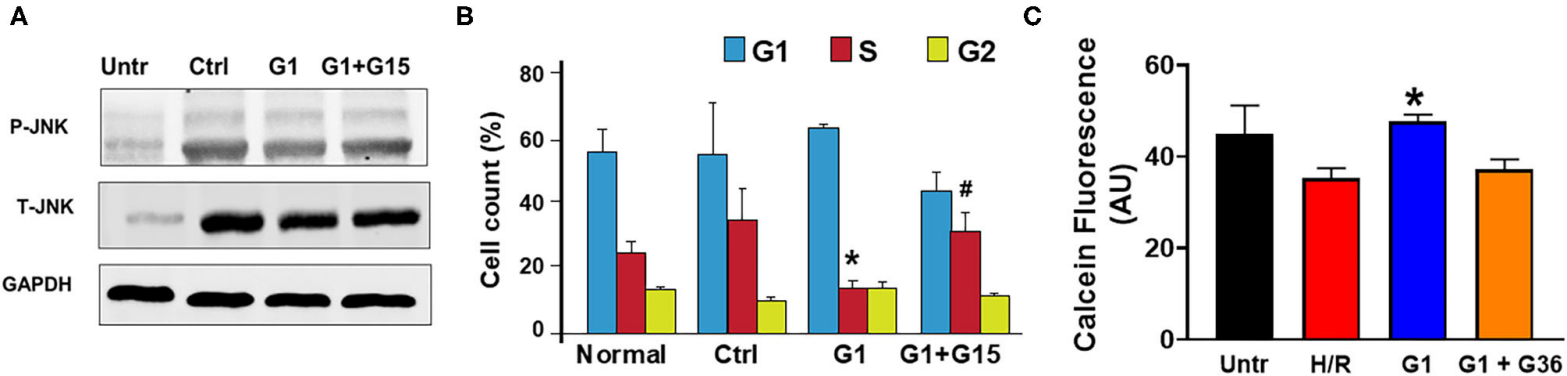
Chronic GPER1 activation protects isolated cardiomyocytes from hypoxia/reoxygenation-induced cell death. **(A)** Representative immunoblots showing a reduction in the expression levels of p-JNK1 and T-JNK1 (total) in the G1-treated cells compared to the Ctrl and G1 + G15. **(B)** Bar graph showing the cell cycle progression analysis assessed by monitoring DNA content with flow cytometry. Relative cell number in each phase was quantified and mean percentage of cells at G1, S, and G2 phases are represented. Note that E2-treated group has fewer cells in the S phase compared to the Ctrl and G1 + G15 groups. Values are expressed as means ± SEM *P < 0.05 vs. Ctrl, ^#^*P* < 0.05 vs. Ctrl, *n* = 6/group. **(C)** Cell viability assay (calcein fluorescence) on isolated adult cardiomyocytes subjected to 6-h hypoxia followed by 48-h reoxygenation (H/R) in media supplemented with no serum (control), G1, or G1 + G36. Note the increase in the fluorescence intensity in G1-treated cells compared to control, and G1 + G36, indicating an increase in cell viability.

Activation of the MST1–JNK pathway has been described to promote cell death (72). We, thus, studied whether chronic GPER1 actions deactivate the MST1–JNK axis in H9c2 cardiomyoblasts treated with H_2_O_2_ agent. Western blot analysis performed in whole-cell lysate after treatment with the control, G1, and G1 + G15 revealed that the levels of phosphorylated JNK (p-JNK) were drastically increased in control (vehicle) vs. H_2_O_2_-untreated cells (Figure 6A). However, the p-JNK levels were reduced in chronic G1-treated cells when compared to the control (Figure 6A). Here also, G1 effects were prevented by G15 (Figure 6A) as the levels of p-JNK in the G1 + G15 group were similar to those in the control. This result suggests that the mechanism of chronic GPER1 actions in H9c2 cells treated with the H_2_O_2_ agent involves deactivation of the MST1–JNK axis.

### Chronic GPER1 Activation Rescues H9c2 Myoblasts From H_2_O_2_-Induced S phase Arrest

We further determined the impact of chronic GPER1 activation on cell cycle progression. We found that G1 treatment prevented H_2_O_2_-induced inhibition of cell proliferation. In fact, our results indicate an accumulation of cells in the S phase following H_2_O_2_ treatment in the control group (35 ± 3.45%) compared to H_2_O_2_-untreated cells (25 ± 0.35%). We observed a slight depletion of cells from the G2/M phase, suggesting that H_2_O_2_ treatment leads to S phase cell cycle arrest and a corresponding decreased cell entry into the G2-M stage (Figure 6B). However, this S phase cell cycle arrest was abrogated by G1 treatment. While control-treated cells had 35% of the cells trapped in the S phase, chronic G1 treatment reduced that level of S phase of cells to 14 ± 2.5% (Figure 6B). However, cotreatment of cells with G1 and G15 failed to rescue cells from the S phase cell cycle arrest (32.44 ± 6). These results suggest that chronic GPER1 actions protect against H_2_O_2_-induced S phase arrest of cell cycle.

### Chronic GPER1 Protects Adult Cardiomyocytes Against Hypoxia Reoxygenation Injury

We, thereafter, determined whether our findings obtained using H9c2 cardiomyoblasts are transposable to other cardiac cell types. To this end, we isolated adult cardiomyocytes from 4- to 6-month-old male Sprague–Dawley rats and subjected them to 6-h hypoxia followed by 48-h reoxygenation. G1 (1 μm), G1 + G36 (100 μm), or vehicle (control) were supplemented in the culture medium at the onset of reoxygenation. The hypoxia/reoxygenation was substituted to H_2_O_2_ treatment for these experiments as this model mimics the *in vivo* ischemia/reperfusion-induced increase in reactive oxygen species (ROS) that is known to increase H_2_O_2_ production by the mitochondria. The cell viability was determined using Calcein assay. We found that G1-treated cardiomyocytes exhibited higher calcein fluorescence intensity compared to the control (vehicle). Here also, supplementation of G36 (another GPER1 antagonist) (73) prevented G1-induced increase in cardiomyocyte viability (Figure 6C). These results indicate that chronic GPER1 activation improves cardiomyocyte survival and viability against hypoxia/reoxygenation-induced cell death.

## Discussion

In this study, we report that chronic GPER1 activation induces cytoprotective effects against H9c2 rat cardiomyoblasts subjected to a cytotoxic H_2_O_2_ agent treatment by preventing the S phase cell cycle arrest, reducing mitochondrial dysfunction, delaying the mPTP opening, and deactivating the MST1/YAP and MST1/JNK pathways.

GPER1 activation is now well-known for inducing protective effects in several disease models, including I/R injury, hypertension (4–6), Parkinson's disease (23), retinal ganglion degeneration (74), and breast cancer (75). In fact, GPER1 activation has been reported to exert protective effects against harmful effects in several other organs, including the heart (4–6), brain (21, 76), muscle (77), testes (77), intestine (26), and kidney (78). Using isolated perfused heart model, others and we have reported that acute (~1 h) pre-ischemic E2 treatment induces cardioprotective effects against I/R injury through GPER1 activation (4–8). Recently, using animals genetically modified subjected to I/R, we have revealed that pre-ischemic E2 treatment induces cardioprotective effects essentially through GPER1 activation and that ERα (Esr1) and ERβ (Esr2) are not needed for this effect (4). We further demonstrated in intact rats (*in vivo*), subjected to LAD artery occlusion followed by reperfusion, that the acute post-ischemic E2 administration induced reduction in myocardial infarct size compared to vehicle effects were abolished by the GPER1-selective antagonist, G15 (5). The acute effects of E2 have been extensively studied more so than the chronic effects. Indeed, chronic activation of E2 has also been shown to improve cell survival after injury (38, 39, 79), but the mechanism by which chronic E2 administration post-stress elicits protective effects remain elusive. In this study, using both H9c2 cardiomyoblasts and adult cardiomyocytes, we found that chronic treatment with the GPER1 agonists G1 or E2 reduces H_2_O_2_- and hypoxia/reoxygenation-induced cell death and reduction in viability compared to vehicle. We reveal that these G1/E2 effects were prevented by the GPER1 antagonists, G15 or G36, suggesting that chronic GPER1 activation induces cytoprotective effects against oxidative stress-induced cell death. This observation is in the line of many studies showing that *in vitro* activation of GPER1 induces cytoprotective effects in different models (80, 81). However, the mechanisms by which chronic GPER1 activation induces cytoprotection is being elucidated because of the assumption that GPER1 activation mediates mostly the rapid action of estrogen.

Lozano and Elledge have reported that cell cycle arrest in response to DNA damage from a variety of stimuli allows time for repair or direct cell apoptosis (82). After DNA damage, the cell cycle is arrested at the transition from G_1_ to S phase or from G_2_ to M phase of the cell cycle. We recently observed that H9c2 cells treated with Mitofilin siRNA have condensed and fragmented nuclei, and this effect was associated with a prolonged S phase of the cell cycle that promotes cell apoptosis (83). We, therefore, postulated that the cytoprotective effect of chronic GPER1 activation could result from its ability to prevent the H_2_O_2_-induced cell cycle arrest. In fact, we found that after H_2_O_2_ treatment, in control-treated cells, 35% of the cells were trapped in the S phase much more than untreated cells indicating that H_2_O_2_ treatment causes S phase cell cycle arrest that decreases cell entry into the G2-M stage. However, after chronic G1 treatment, the level at S phase was reduced to 14% only (Figure 6A). Since the cotreatment of cells with G1 supplemented with G15 failed to rescue cells from the S phase cell cycle arrest, it suggests a mechanism via GPER1 activation.

The role of mitochondria in the generation of cellular energy in normal physiological functions is well-known. Mitochondria are organelles that provide a lot of energy to support cardiac contraction in cardiomyocytes, whereas cardiomyocyte damage can arise as a result of mitochondrial dysfunction (84, 85). Hence, despite their crucial role in cellular function, the mitochondria have also been implicated in the process of cell death (86). We studied whether chronic GPER1 activation might induce cytoprotective effects against oxidative stress-induced cell death by preserving mitochondrial structural integrity and function. Our results indicate that after H_2_O_2_ treatment, chronic incubation of cells with G1/E2 preserves the mitochondrial structure compared to the control. As shown in Figure 2B, the mitochondria from cells treated with E2 display a normal morphology having regular cristae similar to H_2_O_2_-untreated compared to untreated mitochondria in which the cristae were mostly disrupted. We found that chronic E2 treatment preserves the mitochondrial membrane potential from dissipation (MMP) caused by H_2_O_2_ treatment compared to the control, suggesting that chronic G1/E2 treatment protects the mitochondria structure intensity, therefore improving their function (Figure 2A). Opening of the mitochondrial permeability transition pore (mPTP) in cardiac I/R injury because of Ca^2+^ overload and/or increased mitochondrial reactive oxygen species production causes cell death (4). The mPTP opening is currently considered as a crucial event in the mechanism of cell death after I/R (87). We, therefore, determined whether preservation of the mitochondrial structure by chronic G1/E2 treatment leads to the deactivation of the mPTP opening. Our result indicates that chronic E2 treatment delays the opening of the mPTP compared to the vehicle (Figures 3A,B). This result suggests that chronic G1/E2 actions on the mitochondria result in the delay of the mPTP opening, which is consistent with our previous report that acute E2 treatment exerts cardioprotective effects against I/R injury via inhibition of the mPTP opening (4). Because all these G1/E2 effects are prevented by cotreatment with the GPER1 antagonist, G15, suggesting a GPER1-dependent mechanism, our findings, therefore, indicate that chronic GPER1 actions protect H9c2 myoblasts against death by preserving mitochondrial structure and function as well as inhibiting the mPTP opening (Figure 3C).

Mitochondrial quality control depends upon a balance between biogenesis and autophagic destruction (88). Mitochondria are now well-recognized to be able to modulate their morphology by fission and fusion events (89) and that different morphological states are related to multiple physiological and pathophysiological conditions (7). Mitochondrial fragmentation is often linked to mitochondrial dysfunction as this morphological state predominates during elevated stress levels and cell death (8). Mitochondrial fission requires the cytosolic dynamin-related protein 1 (Drp1) (90), while the outer membrane mitofusin (Mfn) 1 and 2 mediate mitochondrial fusion (91). We determined whether the preservation of mitochondrial structure and function was linked to mitochondria dynamics. We found that the levels of both Drp1 and Mfn2 proteins were not changed in the E2-treated group compared to the control (vehicle) (Figure 2C), suggesting that chronic E2 rescue of mitochondrial integrity is not related to mitochondrial dynamics. Because GPER1 has not been found to localize in the mitochondria, its actions on the mitochondria can only be facilitated by subcellular signaling that we will define in future studies.

GPER1 actions have been shown to involve multiple signaling related to cell survival and proliferation such as MEK/ERK, PI3K/Akt, mTOR, and Hippo/YAP pathways (4, 6, 40, 47, 92). In breast cancer cells, the Hippo/YAP/TAZ pathway has been reported as a key downstream signaling branch of GPER1 actions and plays a critical role in breast tumorigenesis (40). The Hippo/YAP pathway has been previously described to play a key role in cardiac development and regeneration (46, 47). YAP is a key effector of the Hippo pathway. Inhibition of YAP phosphorylation is believed to promote YAP nuclear accumulation, where it upregulates its downstream genes. In an *in vitro* model, treatment with bisphenol, which can promote the migration, but not the proliferation of triple-negative breast cancer cells, has been found to activate YAP, and the inhibition of GPER1 attenuated the effects of BPS-induced YAP dephosphorylation (93). Also, the mammalian Ste20-like kinase-1 (MST1), which has been shown to mediate H_2_O_2_-induced cell death (94), is a central player in the Hippo/YAP pathway. Phosphorylation of MST1 in response to harmful stimuli promotes phosphorylation YAP, and its subsequent degradation by 14-3-3, therefore, favoring upregulation of proapoptotic genes. On the contrary, an increase in the cytosolic MST1 levels due to the decrease in its phosphorylation promotes dephosphorylation of YAP, and increases the levels of YAP, facilitating its translocation in the nucleus where its activation production of genes takes place. We, thus, investigated whether chronic GPER1 actions involve regulation of the MST1/YAP axis. We found that G1 treatment reduces the phosphorylation of both MST1 and YAP, resulting in accumulation and translocation of YAP in the nucleus (Figures 4C,D). The involvement of YAP in the mechanism of G1 is confirmed by the significant increase in cell death in the G1 group when YAP was pre-deleted by shYAP treatment (Figure 5C). We also found a G1-induced decrease in YAP phosphorylation that increases the levels of non-phosphorylated YAP, which is associated with the upregulation of prosurvival genes, including CTGF, CYR61, and ANKRD1 as well as the downregulation of genes involved in apoptosis such as PUMA and Bim. We postulate that the deactivation of the MST1/YAP pathway by the reduction of mitochondria dysfunction that might result in less production of free radicals plays a critical role in the mechanism of chronic GPER1 activation-induced cytoprotective effects. However, further investigations are needed to clearly determine the mitochondria-dependent signaling that inhibits the Hippo/YAP pathway. Nevertheless, we favor the opinion that the reduction in the production of mitochondrial ROS is one of the key reasons for MST1 dephosphorylation.

In this paper, we abundantly refer to GPER1 activation effects as “cytoprotective,” which should logically be used if GPER1 activation was performed before the oxidative stress. In our protocol, GPER1 was activated after the oxidative stress insult; therefore, these GPER1 effects would be better qualified as rescue or restorative effects.

## Conclusion

**Figure 7:**
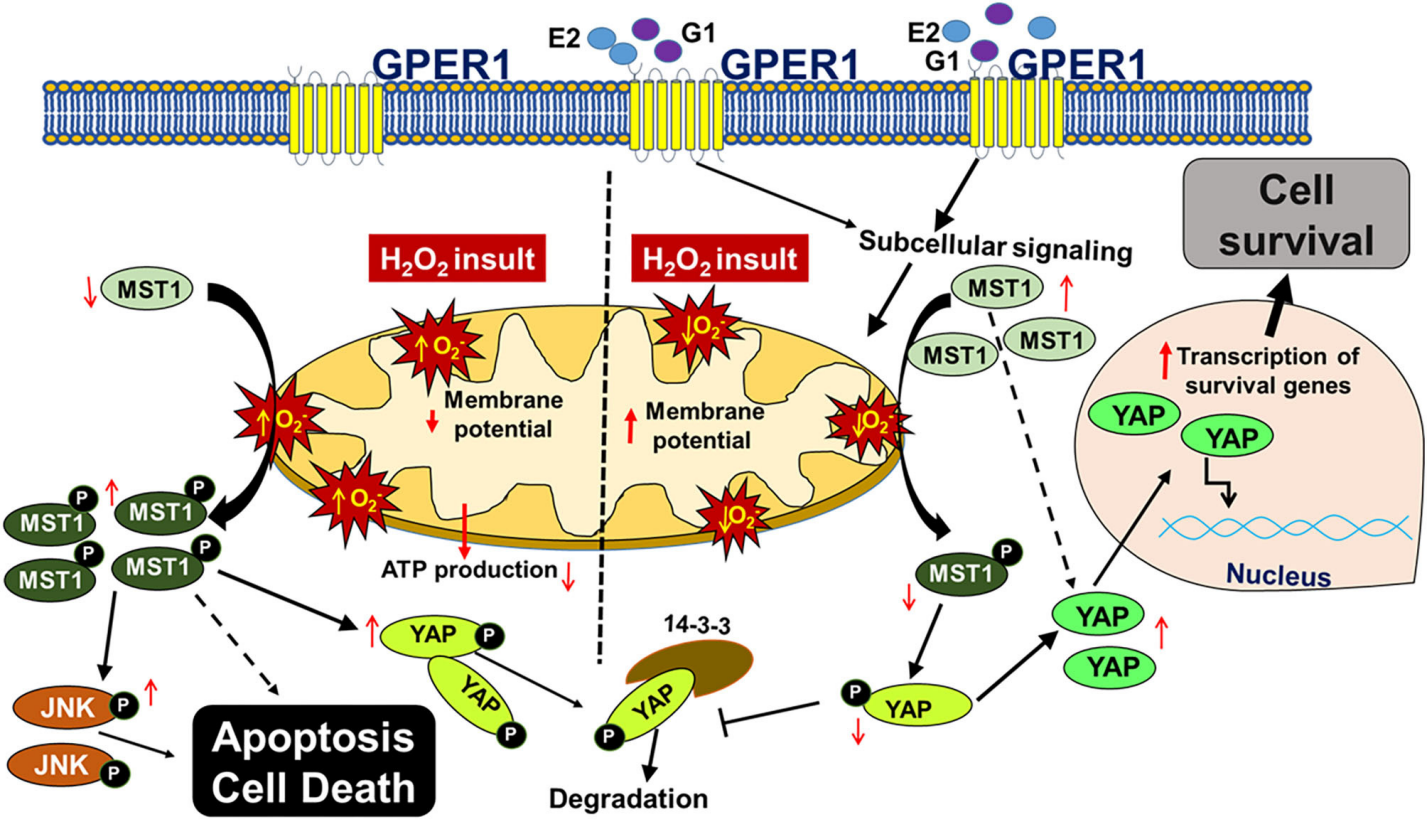
Scheme depicting the mechanism underlying chronic G1-induced cytoprotective effects against H_2_O_2_-induced cell death. In cultured H9c2 myoblasts, treatment with the cytotoxic agent, H_2_O_2_ increases the reactive oxygen species (ROS, ${\text{O}}_{2}^{.}$) production, which promotes the phosphorylation of MST1 (p-MST1). p-MST1 initiates activation of cascades that lead to phosphorylation of YAP (p-YAP). p-YAP is sequestered in the cytosol and subsequently degraded. In addition, increase in p-MST1 promotes the phosphorylation of JNK that upregulates proapoptotic genes. On the other hand, chronic GPER1 activation with its agonists E2 or G1 stimulates subcellular signaling that preserves mitochondrial structure and polarization leading to reduced ROS production and delayed mPTP opening. Reduction in ROS production is postulated to mitigate the phosphorylation of MST1, which increases the non-phosphorylated YAP. Thereafter, YAP translocates into the nucleus to upregulate prosurvival genes that, therefore, promote cell survival.

In this study, we report that chronic activation of GPER1 in H9c2 cardiomyoblasts pretreated with the cytotoxic agent, H_2_O_2_, reduces cell death and enhances cell viability by preserving mitochondrial structural integrity and function that result in the high MMP, the delay of the mPTP opening. Together, these chronic GPER1 activation effects on the mitochondria promote the deactivation of the Hippo/YAP pathway, resulting in the translocation of YAP in the nucleus, where it promotes the upregulation of prosurvival genes and downregulation of proapoptotic genes (Figure 7).

## Data Availability Statement

All datasets generated for this study and included in the article are available from the corresponding author on reasonable request.

## Ethics Statement

The animal study was reviewed and approved by UT Health Science Center at San Antonio Institutional Animal Care and Use Committee (IACUC) institutional.

## Author Contributions

NM, AI, and JB conceived and designed the research. NM, AI, and NT performed the experiments. AI, YF, and JB analyzed the data. AI, NT, YF, and JB interpreted the results of the experiments and edited and revised the manuscript. AI and JB drafted the manuscript. AI, NT, and JB prepared the figures. JB approved the final version of the manuscript. All authors contributed to the article and approved the submitted version.

## Conflict of Interest

The authors declare that the research was conducted in the absence of any commercial or financial relationships that could be construed as a potential conflict of interest.
